# Marine-derived fungi: diversity of enzymes and biotechnological applications

**DOI:** 10.3389/fmicb.2015.00269

**Published:** 2015-04-10

**Authors:** Rafaella C. Bonugli-Santos, Maria R. dos Santos Vasconcelos, Michel R. Z. Passarini, Gabriela A. L. Vieira, Viviane C. P. Lopes, Pedro H. Mainardi, Juliana A. dos Santos, Lidia de Azevedo Duarte, Igor V. R. Otero, Aline M. da Silva Yoshida, Valker A. Feitosa, Adalberto Pessoa, Lara D. Sette

**Affiliations:** ^1^Instituto Latino Americano de Ciências da Vida e da Natureza, Centro Interdisciplinar de Ciências da Vida, Universidade Federal da Integração Latino-AmericanaParaná, Brazil; ^2^Divisão de Recursos Microbianos, Centro Pluridisciplinar de Pesquisas Químicas, Biológicas e Agrícolas, Universidade Estadual de CampinasPaulínia, Brazil; ^3^Laboratório de Micologia Ambiental e Industrial, Departamento de Bioquímica e Microbiologia, Instituto de Biociências, Universidade Estadual Paulista Júlio de Mesquita FilhoRio Claro, Brazil; ^4^Departamento de Tecnologia Bioquímico-Farmacêutica, Faculdade de Ciências Farmacêuticas, Universidade de São PauloSão Paulo, Brazil

**Keywords:** marine-derived fungi, enzymes, marine mycology, culture-dependent methods, culture-independent methods, environmental pollutants, industrial microbiology

## Abstract

The ocean is considered to be a great reservoir of biodiversity. Microbial communities in marine environments are ecologically relevant as intermediaries of energy, and play an important role in nutrient regeneration cycles as decomposers of dead and decaying organic matter. In this sense, marine-derived fungi can be considered as a source of enzymes of industrial and/or environmental interest. Fungal strains isolated from different substrates, such as invertebrates, decaying wood, seawater, sediments, and mangrove detritus, have been reported to be producers of hydrolytic and/or oxidative enzymes, with alginate lyase, amylase, cellulase, chitinase, glucosidase, inulinase, keratinase, ligninase, lipase, nuclease, phytase, protease, and xylanase being among the enzymes produced by fungi of marine origin. These enzymes present temperature and pH optima ranging from 35 to 70^∘^C, and 3.0 to 11.0, respectively. High-level production in bioreactors is mainly performed using submerged-state fermentation. Certain marine-derived fungal strains present enzymes with alkaline and cold-activity characteristics, and salinity is considered an important condition in screening and production processes. The adaptability of marine-derived fungi to oceanic conditions can be considered an attractive point in the field of fungal marine biotechnology. In this review, we focus on the advances in discovering enzymes from marine-derived fungi and their biotechnological relevance.

## Introduction

Marine microbial communities (bacteria, fungi, algae, plankton, and viruses) are considered important ecological components in marine environments due to their performance in biogeochemical processes (Sowell et al., 2008). Marine fungi have been classified as obligate or facultative: obligate marine fungi are those that grow and sporulate exclusively in a marine or estuarine habitat, whereas facultative marine fungi are those from freshwater or terrestrial origin that are able to grow (and possibly sporulate) in marine environments (Kohlmeyer and Volkmann-Kohlmeyer, 2003; Li and Wang, 2009). As a more general classification of these organisms, the term “marine-derived fungi” is often used because most of the fungi isolated from marine samples are not demonstrably classified as obligate or facultative marine microorganisms (Osterhage, 2001).

Many factors can influence the activity, abundance and distribution of fungi in the marine environment. The occurrence of marine fungi has been reported in different substrates (e.g., sponges, algae, wood, tunicates, sediments, mollusks, corals, plants, fish), and the ecology and phylogeny of this group are summarized in Jones (2000), Jones et al. (2009, 2011), Jones and Pang (2012) and Richards et al. (2012). A great diversity of fungi has been recovered from along coastlines, such as mangrove, sand, beach, river, and estuarine habitats, suggesting that environmental influences such as floods and winds carry terrestrial fungi toward marine environments. Thus, marine-derived fungi often routinely exhibit morphological characteristics similar to their terrestrial counterparts (Mejanelle et al., 2000; Morrison-Gardiner, 2002).

A wide range of activities has been identified from marine environment, including antibacterial, antidiabetic, antifungal, anti-inflammatory, antiprotozoal, antituberculosis, antiviral, antitumor, and cytotoxic activities, many of which can be attributed to specific enzymes (Mayer et al., 2013). Although some reports related to enzyme production by marine-derived fungi appeared in the 1980s, studies on this subject started to be published more frequently after 1999–2000 (Velmurugan and Lee, 2012). Salinity, high pressure, low temperature, oligotrophic conditions, pH extremes, widely ranging mineral content in seawater, and special lighting conditions contribute to the differences between the enzymes generated by marine microorganisms and homologous enzymes from terrestrial microorganisms (Booth and Kenkel, 1986; Jones, 2000; Gomes et al., 2008; Madhu et al., 2009; Pang et al., 2011; Intriago, 2012; Passarini et al., 2013; Rämä et al., 2014).

Due to their immense genetic and biochemical diversity, marine microorganisms are viewed as a new promising source of enzymes with potential technological applications (Debashish et al., 2005; Zhang and Kim, 2012). The market for marine fungal enzymes is divided into four segments: (i) technical enzymes, mainly intended for cleaning, textile, leather, biofuel, pulp, and paper industries; (ii) enzymes for food and beverages; (iii) enzymes for animal feed; (iv) enzymes related to environmental applications; and (v) enzymes related to pharmaceutical and cosmetic applications.

The advances in discovering enzymes from marine-derived fungi and their biotechnological relevance are discussed below.

## Production of Enzymes by Marine-Derived Fungi

### Accessing Marine-Derived Fungal Strains

Culture-dependent approaches have served as the main techniques utilized to obtain fungal enzymes for biotechnological applications. However, it is expected that in the near future, molecular applications, such as those based on using recombinant DNA, may be routinely applied for marine enzyme expression directly from metagenomic libraries. Such techniques could allow access to the enzymes produced by microbial communities that are not culturable in the laboratory and allow the discovery of new compounds that can be used in biotechnology, as discussed by Voget et al. (2003) and Singh (2010).

The fungal isolation process begins with substrate sampling. Kjer et al. (2010) reported common methodologies for sample collection and discussed special conditions for the storage of animal and seaweed tissue, and mangrove leaf samples. Surface sterilization of the sample may be performed by repeated washing with sterilized seawater or artificial seawater (ASW). In the study of Menezes et al. (2010), marine invertebrate samples were first sterilized with mercury chloride (in ethanol) and then washed twice with sterilized seawater. In contrast, Passarini et al. (2013) only washed the samples with sterilized seawater, showing an improvement in the rate of fungal recovery. The results from these studies suggest that mercury chloride can reach internal tissues and kill some microbial cells. After surface sterilization, samples can be directly inoculated on agar culture media by plating pieces (1 cm^3^) of the substrate or using the pour-plate technique after substrate trituration and serial dilution.

Different laboratory conditions are applied for the recovery of fungal strains from marine substrates. The greatest diversity can be recovered using poor and rich media, and different temperature, pH, and oxygen conditions. Salinity is an important condition and can be achieved using sterilized seawater or ASW. The culture media typically found in the literature for marine-derived fungal growth are as follows: Glucose Agar, Sabouraud Agar, Boyd, and Kohlmeyer Agar (B&K; Kohlmeyer and Kohlmeyer, 1979), Peptone and Yeast Extract Dextrose Agar, Marine Agar, Potato Dextrose Agar, Malt Agar, Cornmeal Agar, and Oat Agar.

Kjer et al. (2010) described a detailed protocol for the isolation, purification and characterization of endophytic marine-derived fungi, and Sponga et al. (1999) discussed the isolation of fungi from marine sediments and sponges. Hallmann et al. (2006), Abdel-Monem et al. (2013), and Sette and Bonugli-Santos (2013) also mention the use of specific nutrients or compounds with particular toxicity levels for the selection of slow-growing fungi. For yeast isolation, alternative methods have been reported by Brauers et al. (2000) and Schulz et al. (2008), and enrichment methods were applied by Dhevagi and Poorani (2006) and Mohan et al. (2013) for the isolation of marine actinobacteria. However, to our knowledge, there are no reports in the available literature related to enrichment strategies for fungal isolation from marine substrates. Additional information related to the isolation of marine-derived fungi can be found in Vrijmoed (2000) and Nakagiri (2012).

After isolation, fungal strains are purified and preserved, though there is no universal method for microbial preservation because the response to the different methods varies among taxonomic groups and even among strains of the same species. The World Federation for Culture Collections Guidelines (WFCC, 2010), Guidance for the Operation of Biological Research Centers (OECD, 2007), and Guide for the Operation of Cultures Collections from the Brazilian Society of Microbiology (Sette et al., 2007) list a number of guidelines related to the maintenance and preservation of microbiological material. Some factors must be considered when choosing the most appropriate preservation method: (i) the storage period; (ii) the continued viability of the lineage properties; and (iii) the importance of the collection with respect to biodiversity and biotechnological potential. The use of at least two preservation methods is emphasized in all the guidelines cited above; these guidelines also mention issues related to biosecurity and biosafety.

The recommended preservation methods and distribution forms for filamentous fungi and yeasts are summarized in the study reported by Sette et al. (2013) and are listed in **Table 1**.

**Table 1:** Recommended preservation methods for filamentous fungi and yeasts (Sette et al., 2013).

Sporulating strains	Non-sporulating strains
Cryopreservation below -140^∘^C (is preferred)	Under oil or water
Cryopreservation below -80^∘^C (is accepted)	Freeze-drying
Freeze-drying/Liquid-drying	Cryopreservation

Several studies have explored metabolites from marine-derived fungi using a culture-dependent approach (Bugni and Ireland, 2004). In this sense, it is expected that a great number of isolates may be preserved in culture collections from different institutions all over the world, representing a potential source of microbial genetic resources for biotechnological applications. According to Sette et al. (2013), research programs should be put in place as part of culture collection activities, as recommended by WFCC (2010) Guidelines, because it not only helps to attract high-quality staff but can also make an important contribution to the knowledge of the groups of organisms maintained in such collections. In addition, these programs ensure that staff keep abreast of current developments and are aware of the needs of the user community.

It is important to mention that, regardless of the manner in which the microbial genetic resources were obtained (culture-dependent or culture-independent approaches), the access (use) of microbial biodiversity must follow national and international rules, including those established by the Convention on Biological Diversity (CBD, 2011), especially the Nagoya Protocol on Access and Benefit Sharing – ABS.

### Screening and Culture Conditions

There is no single method for screening new enzymes, but preference should be given to the use of low-cost, rapid, and sensitive techniques. When a large number of microorganisms is to be evaluated, high throughput screening (HTS) or medium throughput screening (MTS) methods should be considered in the prospecting stage (Sette et al., 2013). In a somewhat arbitrary definition, ‘medium throughput’ indicates several 100 determinations per day, whereas ‘high throughput’ is currently considered to imply the evaluation of 1,000–10,000 samples per day (Reetz, 2006).

Screening can often begin in the isolation step with the addition of specific nutrient sources (e.g., polyaromatic hydrocarbons – PAHs, pesticides, antimicrobials, inductors) in the culture medium to preferentially select the microorganism able to produce the target enzyme (Da Silva et al., 2004). Colorimetric methods in Petri dishes have been widely used for the selection of microbial enzymes. In these methods, the target enzyme converts a colorless substrate into a colored one or changes the medium color, as used for the screening of asparaginase and glutaminase (Thenmozhi et al., 2011; Patil et al., 2012; Dhale and Mohan-Kumari, 2014), ligninases (Verma et al., 2010; Chen et al., 2011), lipases (Duarte et al., 2013), xylanases, and cellulases (Azad et al., 2013). Analysis of halos, formed by substrate degradation around the colony, may be applied, such as for protease screening (Zhang and Kim, 2010).

During the screening and production of enzymes from marine samples, the presence of salt can be crucial as it is presumed that marine-derived fungal metabolism is adapted to ocean salinity (D’Souza et al., 2006; Chen et al., 2011). Chen et al. (2011) and Bonugli-Santos et al. (2012) achieved better results in lignin-degrading enzyme selection when saline conditions (ASW) were applied. Additionally, Arfi et al. (2013) reported the secretion of salt-adapted lignocellulolytic enzymes from the mangrove fungus *Pestalotiopsis* sp. **Table 2** lists a selection of enzymes produced by marine-derived fungi, growth conditions, and their characteristics; for all enzymes cited, salinity was considered in the culture conditions.

**Table 2:** Enzymes produced by marine-derived fungi: growth conditions and characteristics.

Enzyme	Fungus	Source of isolation (locality)	Growth condition				Enzyme characteristics			Reference
			Temp (^∘^C)	pH	Salinity	Medium	(substrates)	Production	Optimal temp (^∘^C)	Optimal pH	
Protease	*Aureobasidium pullulans*	Saltern sediment (Qingdao, China)	24.5	6.0	Seawater	Soluble starch, NaNO_3_	7,200 U/L	45	9.0	Chi et al. (2007)
Amylase	*Mucor* sp.	Sponge *Spirastrella* sp. (Havelock Island, Andaman Sea, India)	30	5.0	Seawater (50% v/v)	Soluble starch, Casein	41,840 U/L	60	5.0	Mohapatra et al. (1998)
Glucosidase	*Aspergillus* sp. AS 58	Marine sediments (NR)	45	5.0	NR	Pectin	80,000 U/L	60	3.0–4.0	Elyas et al. (2010)
Inulinase	*Pichia guilliermondii* M-30	Algae collected at 100 m depth (Changdao Island, Bohai Sea, Penglai, China)	30	6.5	Solid-state with seawater	Wheat bran, rice bran	455 U/g	60	6.0	Guo et al. (2009)
Laccase	*Cerrena unicolor*	Decaying mangrove wood (Choraõ Island, Goa, India)	30	NR	Half-strength seawater	Modified LN medium	24,000 U/L	70	3.0	D’Souza-Ticlo et al. (2009)
	*Mucor racemosus* CBMAI 847	Cnidarian* Mussismilia hispida* (São Sebastião Coast, São Paulo, Brazil)	28	NR	ASW (2–23% w/v)	Glucose supplemented with wheat bran	898 U/L	NR	5.0	Bonugli-Santos et al. (2010a)
	*Marasmiellus* sp. CBMAI 1062	Sponge *Amphimedon viridis* (São Sebastião Coast, São Paulo, Brazil)	28	NR	ASW (1.0% w/v)	Malt extract	971 U/L	37	5.0	Bonugli-Santos et al. (2010b)
	*Peniophora* sp. CBMAI 1063	Sponge *Amphimedon viridis* (São Sebastião Coast, São Paulo, Brazil)	28	NR	ASW (1.0% w/v)	Malt extract	709 U/L	37	5.0	Bonugli-Santos et al. (2010b)
Lipase (extracellular)	*Geotrichum marinum*	Marine soil (California, USA)	23	NR	Synthetic seawater (38 g/L salt)	Yeast extract, olive oil	NR	40	8.0	Huang et al. (2004)
Lignin peroxidase	*M. racemosus* CBMAI 847	Cnidarian* Mussismilia hispida* (São Sebastião Coast, São Paulo, Brazil)	28	NR	ASW (2–23% w/v)	Glucose supplemented with wheat bran	75,376 U/L	NR	3.0	Bonugli-Santos et al. (2010a)
	*Tinctoporellus* sp. CBMAI 1061	Sponge *Dragmacidon reticulata* (São Sebastião Coast, São Paulo, Brazil)	28	NR	ASW (1.0% w/v)	Malt extract	2,230 U/L	NR	3.0	Bonugli-Santos et al. (2010b)
Manganese peroxidase	*M. racemosus* CBMAI 847	Cnidarian* Mussismilia hispida* (São Sebastião Coast, São Paulo, Brazil)	28	NR	ASW (2–23% w/v)	Glucose supplemented with wheat bran	4,485 U/L	NR	4.5	Bonugli-Santos et al. (2010a)
Xylanase	*Aspergillus niger*	Mangrove leaf detritus (Chorao Island, Goa, India)	28–30	4.5–8.5	Half-strength seawater	Oat spelt xylan, sugarcane bagasse	580 U/L	50	3.5	Raghukumar et al. (2004b)

NR, not reported; ASW, artificial seawater; LN, low nitrogen.

The fungal production of enzymes depends on its physiology, as well as on culture medium composition (Baldrian and Gabriel, 2003). Carbon and nitrogen sources play an important role in enzyme production; their effects have been investigated in numerous fungi from terrestrial environments and are also relevant for marine-derived fungi. Complex substrates, such as starch, casein, pectin, malt extract, wheat bran, olive oil, xylan, and sugarcane bagasse, are used for the production of enzymes by marine-derived fungi (**Table 2**). The enzymes listed in **Table 2** were produced by fungal strains recovered from different substrates, including marine invertebrates (sponges and cnidarian), sediments, algae, decaying wood, and leaf detritus. The optimum temperature and pH of these enzymes ranges from 37 to 70°C and from 3 to 9, respectively.

Additional information on the screening and production of enzymes by marine fungi can be found in Hyde and Pointing (2000), and Velmurugan and Lee (2012).

Because culture conditions clearly influence the production of enzymes, the best way to improve their production is the use of experimental design and statistical analysis. This methodology has been successfully applied to terrestrial fungi (Levin et al., 2008) and provides an efficient approach to determining the best culture conditions for maximizing enzyme production, which in turn can lead to process optimization. Implementing the statistical methodology central composite design, Bonugli-Santos et al. (2010a) concluded that manganese peroxidase production by marine-derived *Mucor racemosus* CBMAI 847 is most likely related to salt concentration. D’Souza-Ticlo et al. (2009) demonstrated the relevance of the use of response surface methodology in the evaluation of the effects and interactions of medium components on laccase production by a marine-derived fungus. In this study, low concentrations of NH_4_Cl and high concentrations of glucose were found to favor the production of biomass.

### Bioprocess

After the optimum culture conditions on a small scale are defined (flasks with shaking), studies related to enzyme production on a larger scale (bioreactors) and scaling-up, which is defined by Bliem and Katinger (1988) as the predictable (engineered) increase in production capacity, must be performed. Substrate consumption, product formation, and cellular biomass are important factors, which should be considered, and quantified, for appropriate scale-up studies. Marine-derived fungal strains adapted to liquid medium may attenuate some of the issues found with terrestrial strains, such as biomass measurement. Additionally, water-adapted strains may show increased enzyme production (Verbist et al., 2000; Masuma et al., 2001).

The large-scale production (e.g., in bioreactors) of glucoamylase, superoxide dismutase, lignin peroxidase, chitinase, protease, and glutaminase by marine strains is reported in the literature (Sarkar et al., 2010). These enzymes are produced in bioreactors largely through submerged-state fermentation, and the conditions related to bioreactor production are listed in **Table 3**.

**Table 3:** Bioprocesses used for the production of enzymes by marine-derived fungi.

Enzyme	Fungus	Source of isolation (locality)	Medium volume (L)	pH	Temp (^∘^C)	Aeration (vvm) / Agitation (rpm)	Time (h)	Carbon source	Enzyme activity	Reference
Amylase	*Aureobasidium Pullulans* N13d	Deep sea (Pacific Ocean)	2.0	4.0	28.0	3.0/250	56	Soluble starch and peptone	40 U/mg	Li et al. (2007a)
Chitinase	*Penicillium janthinellum* P9	NR	2.0	4.0	28.0	1.5/500	240	Colloidal chitin and corn steep liquor	686 U/L	Fenice et al. (1998)
Protease	*Aureobasidium Pullulans*	Saline sediments (Qingdao, China)	2.0	6.0	24.5	4.0/150	30	Soluble starch and NaNO_3_	7 U/mL	Chi et al. (2007)
Superoxide dismutase	*Debaryomyces hansenii* C-11	(Pacific Ocean, Baja, California Sur, Mexico)	1.0	5.0–7.0	30.0	5.0/500	48	Glucose	400 U/mg	Orozco et al. (1998)

Adapted from Sarkar et al. (2010). NR, not reported; Temp, temperature; vvm, volume per volume per minute; rpm, rotation per minute.

Trincone (2011) described an overview of the bioprocess strategies adopted for the cultivation of marine-derived organisms for enzyme production, including protease, chitinase, agarase, and peroxidase. For further discussion about marine enzyme production and novel prospects, see Kristensen et al. (2008) and Trincone (2010).

For many industrial applications, enzymes need to be concentrated, separated, and/or purified from the medium, and the purification strategies employed should be inexpensive, have a high yield and selectivity, be amenable to large-scale operations and should have the potential for continuous product recovery (Gupta et al., 2004; Singh and Mukhopadhyay, 2012). In some cases, purification methodologies need to be adapted to maintain the integrity of the enzymes because catalytic activity is dependent upon conformational structure. Several strategies can be applied to obtain purified enzymes. However, specific studies for establishing strategies of marine enzyme purification are scarce. Some reports of marine-derived fungal enzyme isolation and purification are presented in **Table 4**. The degree of purification varies significantly as a function of the number and order of steps, and the purification process in most cases is based on sequential steps, with low and high resolution. In general, low-specificity methods are employed, such as concentration followed by chromatography. Purification processes applied to enzymes from marine fungal strains (**Table 4**) can reach a recovery yield of 66% and a purification factor up to 647-fold. In most cases, increasing the number of steps leads to greater enrichment, albeit a severe decrease in yield is observed.

**Table 4:** Process conditions applied to purify enzymes from marine-derived fungi.

Enzyme	Fungus	Source of isolation (locality)	Sequential purification steps	Purification parameters		Enzyme characteristics		Reference
				Yield (%)	Purification factor (fold)	Optimal temperature (^∘^C)	Optimal pH	
Alginate lyase	*Aspergillus oryzae*	(Kalubhar Island, Gulf of Kutch, Gujarat, India)	Ammonium sulfate precipitation, dialysis, ion exchange chromatography (DEAE-Cellulose), gel filtration (G-50)	21.1	140.1	35	6.5	Singh et al. (2011)
Amylase	*Mucor* sp.	Deep sea (Pacific Ocean)	Ion exchange chromatography (DEAE-Cellulose, DE52)	NR	NR	60	5.0	Mohapatra et al. (1998)
	*Aureobasidium pullulans* N13d	Sponge *Spirastrella* sp. (Havelock Island, Andaman Sea, India)	Tangential flow filtration, ammonium sulfate precipitation, dialysis, gel filtration (Sephadex G-75), PEG concentration, gel filtration (Sephadex G-75)	58.0	7.3	60	4.5	Li et al. (2007b)
Chitinase	*Plectosphaerella* sp. MF-1	Calcareous shell (Yellow Sea, South Korea)	Ammonium sulfate precipitation, dialysis, ion exchange chromatography (DEAE-Cellulose), gel filtration (Sephadex G-100)	0.9	2.9	37	3.0–4.0	Velmurugan et al. (2011)
Fructosyl-amine oxidase	*Pichia* sp*.* N1-1	Coastal seawater (Izu Peninsula, Shizuoka, Japan)	Dialysis, ultracentrifugation, ion exchange chromatography (DEAE-Toyopearl), lyophilization, gel filtration (TSK-Gel)	NR	NR	NR	NR	Sode et al. (2001)
Fucoidanase	*Dendryphiella arenaria* TM94	Sea sand (Baltic Sea, Germany)	Extraction, acetone precipitation, gel filtration (Sephadex G-100)	17.7	26.7	50	6.0	Wu et al. (2011)
Galactosidase	*Guehomyces pullulans* 17-1	Sea sediments (Antarctica)	Gel filtration (Sephadex G-200), ion exchange chromatography (CM-Sepharose), ultrafiltration	16.1	2.4	50	4.0	Song et al. (2010)
Glucanase	*Williopsis saturnus * WC91-2	Sea (Japan)	Tangential flow filtration, gel filtration (Sephadex G-75), ion exchange chromatography (DEAE-Sepharose)	57.0	115.0	40	4.0	Peng et al. (2009)
	*Chaetomium indicum*	Bottom sediments (South China Sea, China)	Ion exchange chromatography (CM cellulose), rechromatography (CM-Cellulose), gel filtration (Bio-Gel P-200)	1.2	159.0	60	4.4 and 5.6	Burtseva et al. (2003a)
	*Trichoderma aureviride* KMM 4630	Culture Collection (China)	Ultrafiltration, ammonium sulfate precipitation, hydrophobic interaction chromatography (Phenyl-Sepharose), rechromatography (Phenyl-Sepharose), dialysis, ultrafiltration, ion exchange chromatography (15 Q PE), ion exchange chromatography (15 S PE)	1.0-2.0	NR	40	5.2	Burtseva et al. (2003b)
Glucosaminidase	*Penicillium canescens*	Sea (Japan)	Ultrafiltration, gel filtration (Sephacryl S-300), ion exchange chromatography (DEAE-Cellulose), gel filtration (Superose 12HR), rechromatography (Superose 12HR)	2.4	155.0	45	4.5	Burtseva et al. (2010)
Glucosidase	*Penicillium canescens*	Seawater (Kerala coastal areas, India)	Ultrafiltration, gel filtration (Sephacryl S-300), ion exchange chromatography (DEAE-Cellulose), rechromatography (DEAE-Cellulose), gel filtration (Superose 12 HR)	4.0	121.0	70	5.2	Dubrovskaya et al. (2012)
	*Aspergillus sydowii* BTMFS 55	Culture Collection of Marine Microorganisms, Pacific Institute of Bioorganic Chemistry (Russian)	Ammonium sulfate precipitation, ion exchange chromatography (DEAE-Sepharose)	0.9	7.0	50	5.0	Madhu et al. (2009)
Hexosaminidase	*Phoma glomerata*	Culture Collection of Marine Microorganisms, Pacific Institute of Bioorganic Chemistry (Russian)	Ion exchange chromatography (DEAE-Cellulose), ion exchange chromatography (DEAE-Sephacell), gel filtration (Toyopearl HW-50)	35.0	36.4	NR	6.0–8.0	Zhuravleva et al. (2004)
Inulinase	*Cryptococcus* sp. SY3	Sea sediment (South Sea, China)	Ammonium sulfate precipitation, dialysis	NR	NR	NR	NR	Bharathi et al. (2011)
	*Pichia guilliermondii*	Gut of *Lutjanus campechanus* fish (Mahabalipuram coastal areas, India)	Tangential flow filtration, dialysis, gel filtration (Sephadex G-75), ion exchange chromatography (DEAE-Sepharose)	7.3	1.5	60	6.0	Gong et al. (2008)
	*Cryptococcus aureus* G7a	Alga collected at 100 m depth (Changdao Island, Bohai Sea, Penglai, China)	Tangential flow filtration, gel filtration (Sephadex G-75), dialysis, ion exchange chromatography (DEAE-Sepharose)	22.4	7.2	50	5.0	Sheng et al. (2008)
Keratinase (Ahm1)	*Penicillium* sp. Morsy1	Soft coral *Dendronephthya hemprichi* (NR)	Ammonium sulfate precipitation, ion exchange chromatography (DEAE-Sepharose), gel filtration (Sephacryl S-200)	4.9 26.7	3.6 17.5	50 60–65	7.0–8.0 10.0–11.0	El-Gendy (2010)
Laccase	*Cerrena unicolor* MTCC 5159	Decaying mangrove wood (Choraõ Island, Goa, India)	Ultrafiltration, gel filtration (Superdex 75), ion exchange chromatography (Mono-Q)	17.0	33.0	70	3.0	D’Souza-Ticlo et al. (2009)
	*Trematosphaeria mangrovei*	Decaying mangrove wood (Choraõ Island, Goa, India)	Gel filtration (Sephadex G-100)	NR	NR	65	4.0	Atalla et al. (2013)
	Basidiomycete unidentified NIOCC#2a	Decaying wood (Abou Keer, Alexandria, Egypt)	Ultrafiltration, ion exchange chromatography (Resource Q), ultrafiltration	NR	NR	60	3.0 and 6.0	D’Souza et al. (2006)
Lignin peroxidase	*Flavodon flavus*	Decaying leaves of *Thalassodendron ciliatum* grass (Mjimwema creek, Indian Ocean, Dar es Salaam Coast, Tanzania)	Ultrafiltration, gel filtration (Sephadex G-25), ion exchange chromatography (Q-Sepharose)	13.5	8.3	NR	NR	Mtui and Nakamura (2008)
Lipase	*Aspergillus awamori* BTMFW032	Seawater (Arabian Sea, Kerala Coast, India)	Ammonium sulfate precipitation, ion exchange chromatography (DEAE-Cellulose)	33.7	30.2	40	7.0	Basheer et al. (2011)
Nuclease	*Penicillium melinii*	*Ascidium* sp. (near Shikotan Island, Sea of Okhotsk, Russia)	Heat treatment, hydrophobic interaction chromatography (Glycine-chitosan), gel filtration (G-75)	66.0	165.0	75	3.7	Balabanova et al. (2012)
Phytase	*Kodamaea ohmeri* BG3	Gut of *Hexagrammos otakii* fish (NR)	Ammonium sulfate precipitation, dialysis, gel filtration (Superdex 75), dialysis, ion exchange chromatography (DEAE-Cellulose)	10.4	7.2	65	5.0	Li et al. (2008)
Polygalacturonase (p36) Polygalacturonase (p40)	*Cryptococcus liquefaciens* N6	Deep-sea sediment from submersible Shinkai 6500, at a depth of approximately 4500–6500 m (Japan Trench)	Ammonium sulfate precipitation, dialysis, ion exchange chromatography (CM-Toyopearl)	NR	NR	40 50	5.0 5.0	Abe et al. (2006)
Protease	*Aureobasidium pullulans* 10	Saltern sediment (Qingdao, China)	Ammonium sulfate precipitation, gel filtration (Sephadex G-75), ion exchange chromatography (DEAE-Sepharose)	18.8	2.1	45	9.0	Ma et al. (2007)
	*Aspergillus ustus* NIOCC#20	Deep-sea sediments at a depth of approximately 5000 m (Central Indian Basin, India)	Speed vacuum concentrator, ion exchange chromatography (Resource-Q), gel filtration (Superdex-200)	21.0	4.0	45	9.0	Damare et al. (2006)
Xylanase (Xil I) Xylanase (Xil II)	*Aspergillus niger* NIOCC 3	Mangrove detritus (Chorao Island, Goa, India)	Ammonium sulfate precipitation, gel filtration (G-100)	12.5 4.9	104.0 647.0	50 50	3.5 3.5	Raghukumar et al. (2004b)

NR, not reported; Temp, temperature.

Once the purified enzyme is successfully obtained, relevant information can be achieved through physicochemical and biochemical characterization. These characteristics are essential for guiding an effective choice of large-scale purification strategy and realizing the application potential of the enzyme. It is noteworthy that no industrial processes for enzyme purification of marine origin were found in the available literature. Data from the studies cited in **Table 4** show that the temperature and pH optima of the purified enzymes produced by marine-derived fungal strains ranged from 35 to 75°C and 3.0 to 11.0, respectively.

The vast majority of the enzymes cited in **Tables 3** and **4** were obtained from ascomycetes fungi, with a small percentage produced by representatives of basidiomycetes. The predominance of ascomycetes in aquatic habitats has been discussed in the literature, with the major hypothesis for explaining this predominance being the presence of spores with adaptation (appendages) to the aquatic ecosystem (Hyde and Jones, 1989; Prasannarai and Sridhar, 2001; Vijaykrishna et al., 2006). This group represents fungi that are readily cultivable and can be easily recovered when culture-dependent techniques are applied (Baker et al., 2009). Conversely, basidiomycetes fungi are rarely isolated from marine samples (Menezes et al., 2010).

Different species from the genera *Aspergillus* and *Penicillium* are cited as marine-derived producers of enzymes. These fungi are salt-tolerant and have been reported in the literature as invertebrate-inhabiting fungi (Holler et al., 2000; Da Silva et al., 2008; Baker et al., 2009; Menezes et al., 2010). The *Aspergillus* sp. and *Penicillium* sp. cited in **Tables 3** and **4** were recovered from marine invertebrates, seawater, deep sediments, and mangrove detritus.

## Biotechnological Potential

Regarding the ecological role of fungi of marine origin, studies have demonstrated that their main activities are indeed associated with the decomposition of organic matter. Within this context, a great diversity of hydrolytic and oxidative enzymes, which can be used in biotechnological processes, have been reported for different species of marine fungi (**Tables 2–4**).

According to Velmurugan and Lee (2012), marine-derived fungi are able to produce enzymes with novel physiological characteristics, such as high salt tolerance, thermostability, barophilicity, and cold-activity. However, few studies to date have shown that enzymes from marine fungal strains are different from those produced by their terrestrial counterparts (Chi et al., 2009). Alkaline xylanases and thermostable metal-tolerant laccases are produced by marine-derived strains of *Aspergillus niger* and *Cerrena unicolor* (Raghukumar et al., 2004b; D’Souza-Ticlo et al., 2009). Low-temperature active endoglucanases were obtained by several fungal strains from the marine sponge *Haliclona simulans* in Ireland (Baker et al., 2010), and cold-active xylanase was produced by a marine-derived *Cladosporium* sp. (Del-Cid et al., 2014) and by a recombinant marine fungal strain (a psychrotrophic fungus from the Yellow Sea; Hou et al., 2006). Chitinases active at low temperatures (5 and 10°C) were also reported by Fenice et al. (1998) and Velmurugan et al. (2011). Lipases, proteases and cellulases were reported to be produced on solid media at 15°C by Antarctic marine yeast strains isolated from marine samples (e.g., different marine invertebrates and sediments; Duarte et al., 2013).

Cold-active microbial enzymes have attracted increasing attention in recent years (Wang et al., 2012). In addition to the properties related to their structural characteristics, one of the main advantages related to the use of these enzymes is the decrease energy expenditure and processing costs associated with industrial heating steps (Duarte et al., 2013). Salt-tolerant fungi and their salt-tolerant enzymes (mainly lignin-degrading enzymes) have been used for bioremediation of environmental pollutants (Passarini et al., 2011), as described in Section “Environmental Applications.” The discovery of barotolerant enzymes is still in the initial phase (Velmurugan and Lee, 2012).

The potential ability of marine-derived fungi to grow on relatively rather simple and inexpensive substrates, and produce enzymes with different physiological characteristics can place them at the forefront of contemporary commercial applications.

### Environmental Applications

#### Decolorization of Synthetic Dyes and Textile Eﬄuents

Residual dyes from different sources introduce organic pollutants into natural water resources or wastewater treatment systems (Zaharia and Suteu, 2012). These dyes belong to classes of compounds with azo, anthraquinone, triphenylmethane, and heterocyclic polymeric structures. According to Diwaniyan et al. (2010), azo dyes are the largest and most versatile class of dyes and account for more than half of the annually produced synthetic dyes.

In aquatic ecosystems, dyes can interfere with photosynthesis and the diffusion of gasses and are of human health concern (Baughman and Weber, 1994; Ciullini et al., 2008). Furthermore, these compounds are often recalcitrant, and their removal from wastewater is difficult and expensive (Hao et al., 2000). In this regard, considerable effort has focused on developing efficient and cost-effective technologies for treating wastewater dyes, including bioremediation, a process in which biological agents are used to degrade environmental pollutants. Bioremediation is based on the exploration of microbial populations that can modify or decompose certain pollutants (Peixoto et al., 2008). Microorganisms used in remediation can be considered as an attractive biotechnological alternative for achieving possible mineralization of the pollutant and its transformation into less toxic products with greater solubility in water, which can then be degraded by the action of other microorganisms (Cerniglia, 1997; Cerniglia and Sutherland, 2001).

Several fungi are known to be capable of degrading persistent pollutants (Haritash and Kaushik, 2009), including textile dyes. Because a large number of textile processes can generate eﬄuents having saline and alkaline conditions, fungi from marine environments demonstrate an important biological advantage for eﬄuent decolorization/degradation because these fungi are adapted to high salt and pH. The dye decolorization mechanism by fungal cells includes oxidative reactions, which can generate non-toxic derivatives (Ciullini et al., 2008). Among the extracellular enzymes produced by filamentous fungi, the ligninolytic system is of great relevance in environmental remediation (Arun et al., 2008).

Raghukumar et al. (1996, 2004a, 2008) and D’Souza et al. (2006) showed significant decolorization of textile eﬄuents and synthetic dyes (e.g., Congo red, Brilliant green, and RBBR) by marine-derived fungi. Other research groups have been focused on the use of marine-derived filamentous fungi for synthetic dye decolorization (Junghanns et al., 2008; Bonugli-Santos et al., 2012; Chen et al., 2014). In the study conducted by Chen et al. (2014), a whole-cell immobilization system (using marine-derived fungi *Pestalotiopsis* sp. J63 and *Penicillium janthinellum* P1) showed the ability to decolorize Azure B dye.

Nutrients and physical parameters have a significant effect on dye decolorization (Singh et al., 2013), and the mechanism involving laccase can differ depending upon the dye structure (D’Souza et al., 2006). According to Verma et al. (2010), marine-derived fungi were able to decolorize two textile eﬄuents: TEA (containing an azo dye with a pH of 8.9) and TEB (containing a mixture of eight reactive dyes with a pH of 2.5). The ascomycetes and basidiomycetes studied presented 30 to 60% TEA decolorization and 33 to 80% TEB decolorization, respectively, under saline conditions. Additional analyses of toxicity (measured by LC50 values against *Artemia* larvae) and a mass spectrometric scan of eﬄuents after fungal treatment revealed degradation of most of the eﬄuent components. The better capacity of marine-derived basidiomycetes to decolorize and degrade textile dyes corroborates the results of many studies cited in literature using terrestrial basidiomycetes fungi. Indeed, basidiomycetes are considered the best producers of ligninolytic enzymes, mainly those classified as white-rot fungi.

Sponge-derived basidiomycetes showed the ability to decolorize textile dyes in solid medium under both saline and non-saline conditions (Bonugli-Santos et al., 2012). Additionally, complete RBBR decolorization was reached in liquid medium, with the best decolorization obtained using *Tinctoporellus* sp. CBMAI 1061 after 3 days of incubation at two concentrations of RBBR (500 and 1,000 mg/L). RBBR was also reported to be degraded by filamentous fungi isolated from scleractinian coral and zoanthids collected along the north coast of São Paulo State, Brazil. In another study, *Penicillium citrinum* CBMAI 853 was the most efficient fungus, decolorizing RBBR (100%) after 12 days, followed by *A. sulphureus* CBMAI 849 (95%), *Cladosporium cladosporioides* CBMAI 857 (93%) and *Trichoderma* sp. CBMAI 852 (89%; Da Silva et al., 2008).

According to Raghukumar et al. (2004a), marine-derived fungi are often more effective than terrestrial fungi in the treatment of various colored eﬄuents because they are better adapted to perform under extreme conditions (high salinity).

#### Degradation of Polycyclic Aromatic Hydrocarbons (PAHs)

Polycyclic aromatic hydrocarbons (PAHs) are widely distributed in the environment and may persist for extended periods of time (Shuttleworth and Cerniglia, 1995). PHA molecules are composed of two or more fused benzene rings and are formed during the combustion of organic molecules and its subsequent recombination (Haritash and Kaushik, 2009). Forests, oil seeps, volcanic eruptions and exudates from trees constitute some natural sources of PAHs. Anthropogenic sources of PAH include fossil fuel burning, coal tar, wood, garbage, refuse, waste lubricating oil, and oil filters, municipal solid waste incineration and petroleum spills and discharge (Kaushik and Haritash, 2006). Certain PHAs are considered toxic, mutagenic, and carcinogenic (Peakall et al., 1982).

The basis for the various known mechanisms of the aerobic metabolism of PAHs involves the oxidation of the aromatic ring (Bamforth and Singleton, 2005). The ligninolytic system and the monooxygenase system of cytochrome P-450 may be involved in PAH degradation by filamentous fungi (Haritash and Kaushik, 2009).

Passarini et al. (2011) reported that the fungus *A. sclerotiorum* CBMAI 849 showed 99.7% pyrene (2 mg in 30 mL) and 76.6% of benzo[a]pyrene (1 mg in 30 mL) degradation after 8 and 16 days, respectively. Benzo[a]pyrene depletion (>50.0%) was also achieved by *Mucor racemosus* CBMAI 847. HPLC-DAD-MS data showed that *A. sclerotiorum* CBMAI 849 and *M. racemosus* CBMAI 847 are able to metabolize pyrene to pyrenylsulfate and benzo[a]pyrene to benzo[a]pyrenylsulfate, suggesting that the mechanism of hydroxylation is mediated by a cytochrome P-450 monooxygenase, followed by conjugation with sulfate ions. In the study performed by Wu et al. (2009), *Aspergillus* sp. BAP14 isolated from marine sediment (the China coast) showed the ability to degrade benzo[a]pyrene: the fungus was able to remove approximately 30% and 60% BaP (0.010 mg/mL) after 3 and 12 days, respectively. In another study, two non-identified marine-derived fungi (NIOCC#312 and NIOCC#2a) were able to remove phenanthrene from a culture medium by adsorption on the fungal mycelium (Raghukumar et al., 2006).

Considering that the use of marine-derived fungi for the bioremediation of polluted saline environments is facilitated by their tolerance to saline conditions, these microorganisms are important microbial resources for biotechnological application in the bioremediation of PAH-polluted environments, such as ocean and marine sediments.

### Industrial Applications

Different enzymes produced by marine-derived fungi have been reported in the literature and are related to the industrial production of: (i) lipases, for the development of cosmetics and as components of medicine (digestive enzymes) or clinical reagents (Zhang and Kim, 2010; Murray et al., 2013); (ii) proteases, for the production of digestive and anti-inflammatory drugs (Zhang and Kim, 2010); (iii) ligninases, with biotechnological applications in many sectors, including such industries as the chemical, fuel, food, agricultural, paper, textile, and cosmetic (Raghukumar et al., 1994; Sette and Bonugli-Santos, 2013); and (iv) others compounds (e.g., L-glutaminase, tannase, and alginase), with potential application in the pharmaceutical and food/beverage sectors (Velmurugan and Lee, 2012).

The proportion of enzymes utilized for food and beverages is constantly growing, with an above-average growth forecast for the next years due to the demand for new applications in the dairy and baking sectors, among others. Studies have highlighted the advances in food technology and have noted marine microorganism capabilities in the production of active compounds, including proteins, and enzymes (Basheer et al., 2011; Dewapriya and Kim, 2014).

Ligninolytic enzymes present important biotechnological properties, since they might be able to degrade a wide variety of substrates via free radical-mediated oxidizing reactions. These enzymes can also be considered a great resource in the biofuel field, due to the possible resistance and activity in the presence of solvents and different pH conditions. Although there are no reports in the available literature related to the use of marine-derived fungi or their enzymes for ethanol production (second generation), Raghukumar et al. (2004b) showed efficient lignin mineralization by the basidiomycete fungus NIOCC#312 isolated from decaying sea grass. Additionally, Intriago (2012) reported the prospect of utilizing marine microorganisms in cellulosic ethanol production. It is important to highlight that fungi classified as basidiomycetes are the best producers of ligninolytic enzymes; therefore, this class of fungi should be considered as the target in studies related to industrial and environmental applications (Chung et al., 2000), including the biological treatment of lignocellulosic substrate for biofuel production.

Despite the relevance of marine-derived fungal enzymes, available data concerning requests or deposits of patents associated with biotechnology using these fungi are lacking. The patents related to marine biotechnology filed in the World Intellectual Property Organization (WIPO) database are mostly associated with bacteria and cyanobacteria.

For data collection, a search was performed in four databases: European Patents Office (EPO), Espacenet-LatPat (Latin, America, and Spain), Industrial Property National Institute (INPI; Brazil), and Bioprospecting Information Resource (United Nations University, Japan). Of these, only EPO afforded results when using the keywords “marine fungi” and “enzyme from marine fungi.” Fourteen filed patents were found, but only eleven are related to biotechnology; none of the registered patents is related to enzyme production.

Another search related to patents based on marine organisms was performed in Bioprospecting Information Resource (database) using a general search (it was performed only in this database due to its smaller size). A total of 105 patents (or requests) were found. The results showed only one German patent related to an unidentified filamentous fungus associated with the marine sponge *Xestospongia exigua*, which is used for the synthesis of biomolecules with pharmaceutical properties. Additionally, a Japanese patent was found related to pectinase production by the yeast *Cryptococcus* sp. The search revealed a low number of marine-derived fungal patents, with 46.6% of the patents (or requests) being related to other marine microorganisms, such as bacteria and archaea. In a previous survey conducted by Teixeira et al. (2010) using the Brazilian database (INPI), 39 patents were found, with 84% referring to algae, 9% to animals, 3% to various microorganisms and 3% to other organisms (non-specified).

**FIGURE 1:**
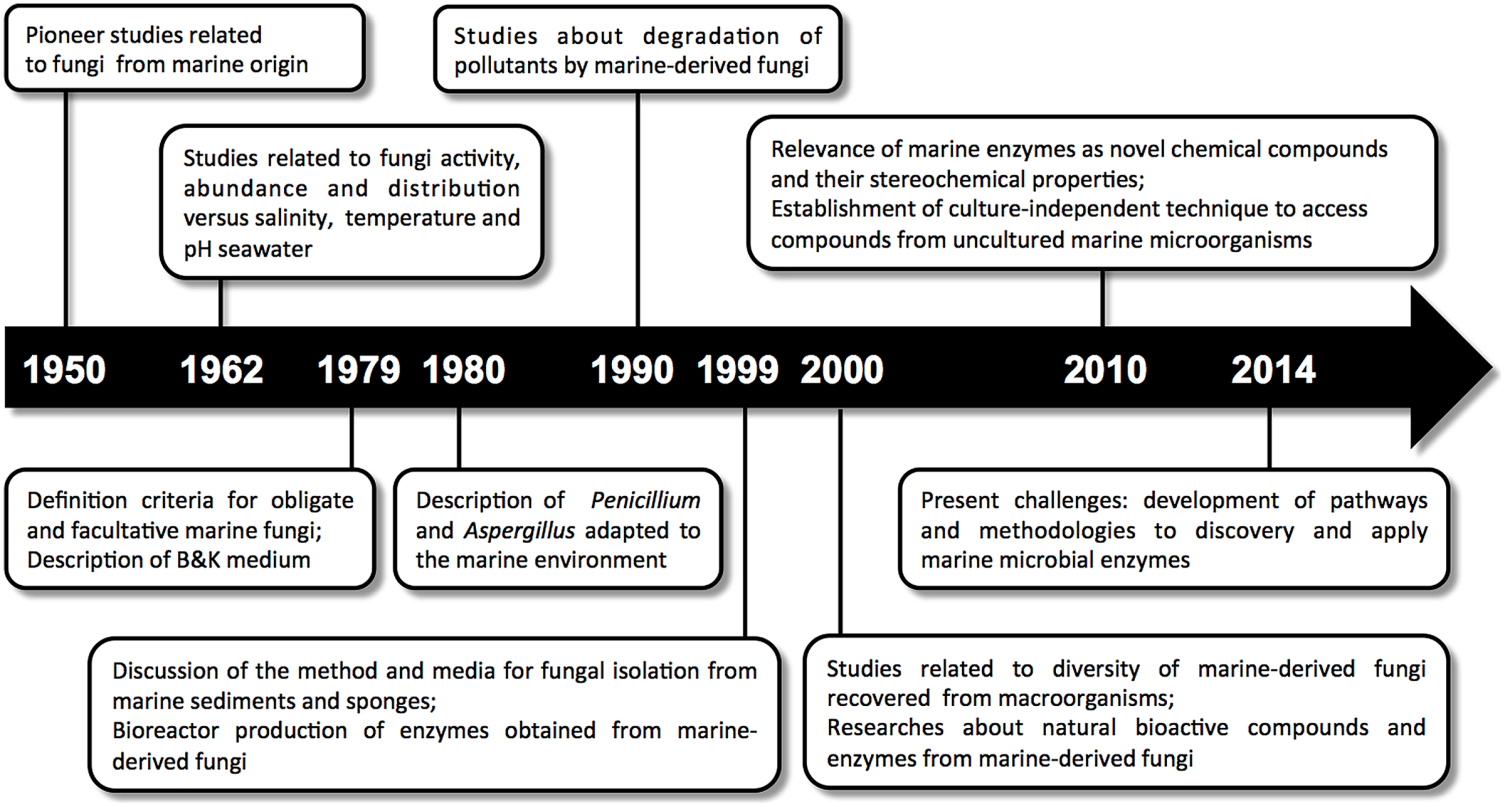
**Timeline of marine mycology: a brief account of the relevant scientific events related to marine-derived fungi and their enzymes**.

Among the marine organisms with biotechnological potential, there are comparatively few requests and patent records for fungi. However, considering the timeline related to the field of marine mycology (**Figure 1**), including the current advances in this area, a significant increase in patent applications should be observed in a near future. In 2014, our research group deposited a patent request at INPI (INPI deposit number BR 10 2014 008502 5) related to the process of laccase enzyme production by the marine-derived basidiomycetes *Peniophora* sp. CBMAI 1063 (laccase enzyme and its use). This enzyme is highly produced only under saline conditions, clearly showing the influence of the marine environment on the production of this enzyme.

## Conclusion and Future Perspectives

Studies related to the prospecting of marine-derived fungal enzymes could result in the discovery of new enzymes that are different from their terrestrial counterparts and also increase our understanding about the diversity and ecology of this microbial group. Taking into account that marine ecosystems are considered a poorly explored environment, and in light of the ongoing studies related to marine-fungal diversity based on culture-dependent and independent approaches, it is reasonable to expect that new fungal taxa recovered from marine habitats will be reported in the short term.

Marine conditions (e.g., salinity, pressure, temperature, and light) contribute to the significant differences between the enzymes produced by marine microorganisms and homologous enzymes from their terrestrial counterparts. However, studies related to the effective difference of these enzymes have not been thoroughly evaluated. Such studies will support applications, and augment our understanding of the ecology, of marine-derived fungi. Molecular characterization, crystallography, and enzyme modulation combined with classical enzymology assessment could assist in addressing questions related to catalyzes and functions. Additionally, studies related to gene transfer should be encouraged to accelerate the development of economically viable biotechnologies associated with the application of marine-derived fungi in the industrial and environmental sectors.

The results presented in this review highlight the potential of marine-derived fungal enzymes for biotechnology. To improve access to marine microorganisms and the use of their enzymes, national and international programs should be established, including the provision of facilities for marine microbial sampling (especially in extreme environments, such as the deep sea); cultivation; prospecting; preservation; and maintenance of culture collections.

## Conflict of Interest Statement

The authors declare that the research was conducted in the absence of any commercial or financial relationships that could be construed as a potential conflict of interest.
